# Prevalence of overweight and obesity in Saudi children and adolescents

**DOI:** 10.4103/0256-4947.62833

**Published:** 2010

**Authors:** Mohammad I. El Mouzan, Peter J. Foster, Abdullah S. Al Herbish, Abdullah A. Al Salloum, Ahmad A. Al Omer, Mansour M. Qurachi, Tatjana Kecojevic

**Affiliations:** a Form the Department of Pediatrics, King Saud University, Saudi Arabia; b From the School of Mathematics, Manchester University, United Kingdom; c From the The Children's Hospital, King Saud Medical Complex, Riyadh, Saudi Arabia; d From the Depatment of Pediatrics, Al-Yamama Hospital, Riyadh, Saudi Arabia

## Abstract

**BACKGROUND AND OBJECTIVE:**

There is limited information on overweight and obesity in Saudi children and adolescents. The objective of this study was to establish the national prevalence of overweight and obesity in Saudi children and adolescents.

**METHODS:**

The 2005 Saudi reference data set was used to calculate the body mass index (BMI) for children aged 5 to 18 years. Using the 2007 WHO reference, the prevalence of overweight, obesity and severe obesity were defined as the proportion of children with a BMI standard deviation score more than +1, +2 and +3, respectively. The 2000 CDC reference was also used for comparison.

**RESULTS:**

There were 19 317 healthy children and adolescents from 5 to 18 years of age, 50.8% of whom were boys. The overall prevalence of overweight, obesity and severe obesity in all age groups was 23.1%, 9.3% and 2%, respectively. A significantly lower prevalence of overweight (23.8 vs 20.4; *P*<.001) and obesity (9.5 vs 5.7; *P*<.001) was found when the CDC reference was used.

**CONCLUSIONS:**

This report establishes baseline national prevalence rates for overweight, obesity and severe obesity in Saudi children and adolescents, indicating intermediate levels between developing and industrialized countries. Measures should be implemented to prevent further increases in the numbers of overweight school-age children and adolescents and the associated health hazards.

Obesity-associated diseases are now reported with increasing frequency in obese children and adolescents. Among these diseases are impaired glucose tolerance, type 2 diabetes mellitus,^1^–^3^ and cardiovascular diseases,^4^ along with other problems such as impaired quality of life,^5^ poor self-esteem,^6^ and depression.^7^ Furthermore, overweight preschool children are more likely to be overweight schoolchildren,^8^ and obese children and adolescents are more likely to become obese adults with all the associated health hazards.^9^,^10^

Surveillance data indicate a high prevalence of overweight and obesity in children from both developed and developing countries. In the United States, the prevalence of overweight (defined as a body mass index [BMI] >85th percentile) and obesity (defined as a BMI >97th percentile) was 31.9% and 11.3%, respectively, for the age group 2 to 19 years of age in the period of 2003-2006 (the prevalence of BMI >95th percentile was 16.3%).^11^ Similarly, 1996 reports from the United Kingdom indicated a 17% prevalence of obesity (BMI >95%) in 15-year-olds,^12^ but in 2004, prevalence rates of 24% and 26% were reported in 11-to 15-year-old boys and girls, respectively.^13^ Reports from developing countries show disparity in the prevalence of overweight and obesity. A high prevalence, similar to that in developed countries, has been reported from countries like Mexico,^14^ Brazil,^15^ Qatar,^16^ the United Arab Emirates,^17^ and Kuwait.^18^ In contrast, a low prevalence has been reported from India (4.9% in 2003 and 6.6% in 2005 for overweight)^19^ and Yemen (6.2% for overweight and and 1.8% for obesity in 2002-2003 for school children).^20^ In Saudi Arabia, reports on the prevalence of overweight and obesity were limited to male school children and a single city.^21^–^23^ To our knowledge, there is no information on the national prevalence of overweight and obesity in Saudi children and adolescents. The objective of this study was to establish the prevalence of overweight and obesity in a representative sample of Saudi school-age children and adolescents.

## METHODS

The prevalence of overweight and obesity in Saudi school-age children and adolescents (5-18 years of age) was calculated from the 2005 growth charts data set from the children and adolescents survey.^24^ Briefly, multistage probability sampling procedures were used to randomly select a cross-sectional sample from a stratified listing based on the population census in Saudi Arabia that was available at the time of the study design. Therefore, the sample was representative of all the socioeconomic strata. Data collection was made by house-to-house visits and a survey questionnaire, clinical examination and body measurements were completed by primary care physicians and nurses. Weight and stature measurements were performed by trained physicians and nurses according to recommended standards.^25^ BMI was calculated according to the formula (weight/height^2^). Percentiles and z scores for age were constructed and smoothed out using the LMS methodology.^26^–^28^ The BMI distribution for Saudi children and adolescents allowed for the calculation of the proportion of children and adolescents whose BMI was above the upper cut-off values.^29^ The prevalence of overweight and obesity were defined using the World Health Organization (WHO) cut-off values as the proportion of children whose BMI for age was above +1 and +2 standard deviation scores (SDS, z scores) respectively. In addition, the prevalence of severe obesity (BMI >+3 SDS) was calculated to estimate the proportion of affected children and adolescents who might be candidates for bariatric surgery. In addition to the 2007 WHO reference, prevalence data were calculated using the widely used 2000 Center for Disease Control (CDC) reference for comparison. All calculations were performed using the software published by the WHO.^30^ Similar calculations were performed using the CDC software whose cut-off values were BMI >85th percentile for overweight and >95th percentile for obesity.^31^ The chi-square test was used to compare proportions and *P*<.05 was considered statistically significant.

## RESULTS

**Figure 1:**
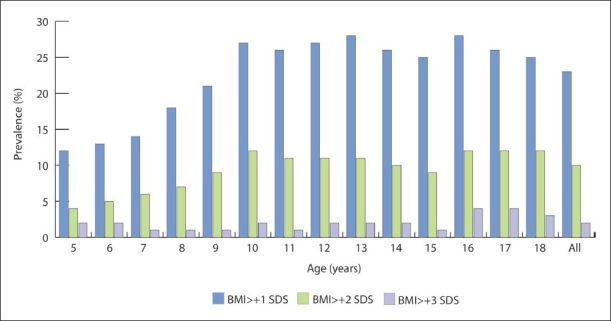
Prevalence of overweight, obesity and severe obesity by age in Saudi children and adolescents from a national sample.

The national sample size in the Saudi reference was 19 317 healthy children and adolescents from 5 to 18 years of age, 50.8% of whom were boys. The prevalence of overweight, obesity and severe obesity in all age groups was 23.1%, 9.3% and 2%, respectively, with boys having a significantly higher prevalence of obesity (10.1% vs 8.4%; *P*<.001) and severe obesity (2.3% vs 1.6%; *P*<.001) than girls (Table 1, Figure 1). However, girls had a significantly higher prevalence of overweight (23.8% vs 22.4%; *P*=.014). In addition, there was a significantly higher prevalence of overweight (26.6% vs 19.5%; *P*<.001), obesity (10.5% vs 8.4%; *P*<.001) and severe obesity (2.4% vs 1.5%; *P*<.001) in adolescents than in school-age children. The prevalence of overweight, obesity, and severe obesity in school-age children (5-12 years) was 19.6%, 7.9% and 1.5%, respectively (Table 2). There was no significant difference in the prevalence of overweight between boys and girls (19.9% vs 19.2%; *P*=.507), but boys had a significantly higher prevalence of obesity and severe obesity (9% vs 6.8%; *P*<.001) and (2% vs 1%; *P*<.001) respectively. The prevalence of overweight, obesity, and severe obesity in adolescents from 13 to 18 years of age was 26.6%, 10.6% and 2.4%, respectively (Table 3). More girls tended to be significantly overweight than did boys (28.4% vs 24.8%; *P*<.001), but there was no significant difference between boys and girls in the prevalence of obesity (11.2% vs 10.0%; *P*=.101) and severe obesity (2.6% vs 2.1%; *P*=.100). Tables 4 and 5 show a significantly lower prevalence of overweight (23.9% vs 20.4%; *P*<.001) and obesity (9.5% vs 5.7%; *P*<.001) when the CDC reference was used.

**Table 1:** Prevalence of overweight and obesity.

Age (years)	BMI > +1 SDS Number of children (%)			BMI > +2 SDS Number of children (%)			BMI > +3 SDS Number of children (%)		
	Boys	Girls	All	Boys	Girls	All	Boys	Girls	All
5–12	6149 (19.9)	5917 (19.2)	12066 (19.6)	6149 (9)	5917 (6.8)	12 066 (7.9)	6149 (2)	5917 (1)	12 066 (1.5)
13–18	3659 (24.8)	3592 (28.4)	7251 (26.6)	3659 (11.2)	3592 (10.0)	7251 (10.6)	3659 (2.6)	3592 (2.1)	7251 (2.4)

**Overall**	**9808 (22.4)**	**9509 (23.8)**	**19 317 (23.1)**	**9808 (10.1)**	**9509 (8.4)**	**19 317 (9.3)**	**9808 (2.3)**	**9509 (1.6)**	**19 317 (2.0)**

SDS: standard deviation scores

**Table 2:** Prevalence of overweight and obesity in school-age children.

Age (years)	BMI > +1 SDS Number of children (%)			BMI > +2 SDS Number of children (%)			BMI > +3 SDS Number of children (%)		
	Boys	Girls	All	Boys	Girls	All	Boys	Girls	All
5	751 (12.3)	700 (10.9)	1451 (11.6)	751 (4.4)	700 (4.3)	1451 (4.4)	751 (1.6)	700 (0.9)	1451 (1.3)
6	757 (12.2)	735 (14.1)	1492 (13.2)	757 (5.3)	735 (4.1)	1492 (4.7)	757 (1.8)	735 (1.1)	1492 (1.5)
7	789 (12.7)	779 (14.8)	1568 (13.8)	789 (6.5)	779 (4.5)	1568 (5.5)	789 (1.8)	779 (0.9)	1568 (1.4)
8	807 (17.1)	751 (18.8)	1558 (18)	807 (8.1)	751 (5.6)	1558 (6.9)	807 (2)	751 (0.7)	1558 (1.4)
9	781 (22.5)	722 (19.1)	1503 (20.8)	781 (10.6)	722 (5.7)	1503 (8.2)	781 (1.7)	722 (1)	1503 (1.4)
10	781 (27.1)	816 (26.1)	1597 (26.6)	781 (13.3)	816 (10.8)	1597 (12.1)	781 (2)	816 (1.8)	1597 (1.9)
11	756 (27.1)	705 (23.8)	1461 (25.5)	756 (11.4)	705 (10.1)	1461 (10.8)	756 (1.7)	705 (0.4)	1461 (1.1)
12	727 (27.9)	709 (26)	1436 (27)	727 (12.2)	709 (9.2)	1436 (10.7)	727 (3.2)	709 (1)	1436 (2.1)

**Total**	**6149 (19.9)**	**5917 (19.2)**	**12 066 (19.6)**	**6149 (9)**	**5917 (6.8)**	**12 066 (7.9)**	**6149 (2)**	**5917 (1)**	**12 066 (1.5)**

**Table 3:** Prevalence of overweight and obesity in adolescents.

Age (years)	BMI > +1 SDS Number of children (%)			BMI > +2 SDS Number of children (%)			BMI > +3 SDS Number of children (%)		
	Boys	Girls	All	Boys	Girls	All	Boys	Girls	All
13	747 (26.1)	680 (31.5)	1427 (28.7)	747 (10.0)	680 (10.9)	1427 (10.5)	747 (1.6)	680 (2.1)	1427 (1.8)
14	720 (24.2)	689 (27.0)	1409 (25.6)	720 (10.3)	689 (10.0)	1409 (10.2)	720 (1.5)	689 (1.9)	1409 (1.7)
15	630 (25.6)	608 (25.2)	1238 (25.4)	630 (11.1)	608 (7.7)	1238 (9.4)	630 (1.7)	608 (1.0)	1238 (1.4)
16	625 (27.0)	616 (30.7)	1241 (28.8)	625 (11.5)	616 (11.4)	1241 (11.5)	625 (3.4)	616 (3.2)	1241 (3.3)
17	521 (22.8)	529 (29.1)	1050 (26.0)	521 (12.1)	529 (10.8)	1050 (11.5)	521 (3.6)	529 (3.0)	1050 (3.3)
18	416 (23.1)	470 (26.8)	886 (25.1)	416 (12.0)	470 (8.9)	886 (10.5)	416 (3.6)	470 (1.5)	886 (2.5)

**Total**	**3659 (24.8)**	**3592 (28.4)**	**7251 (26.6)**	**3659 (11.2)**	**3592 (10.0)**	**7251 (10.6)**	**3659 (2.6)**	**3592 (2.1)**	**7251 (2.4)**

**Table 4:** Comparative prevalence of overweight using the WHO and CDC references.

Age (years)	2007 WHO Number of children (%)			2000 CDC Number of children (%)		
	Boys	Girls	All	Boys	Girls	All
5–12	6149 (19.9)	5917 (19.2)	12 066 (19.6)	5460 (15.9)	5985 (15.7)	11 445 (15.8)
13–18	3659 (24.8)	3592 (28.4)	7251 (26.6)	3253 (24.7)	3127 (25.1)	6380 (24.9)

**Total**	**9808 (22.4)**	**9509 (23.8)**	**19 317 (23.9)**	**8713 (20.3)**	**9112 (20.4)**	**17 825 (20.4)**

**Table 5:** Comparative prevalence of obesity using the WHO and CDC references.

Age (years)	2007 WHO Number of children (%)			2000 CDC Number of children (%)		
	Boys	Girls	All	Boys	Girls	All
5–12	6149 (9)	5917 (6.8)	12066 (7.9)	6260 (5)	5985 (3.9)	12 245 (4.5)
13–18	3659 (11.2)	3592 (10)	7251 (10.6)	3253 (8.2)	3127 (5.5)	6380 (7)

**Total**	**9808 (10.6)**	**9509 (8.5)**	**19 317 (9.3)**	**9513 (6.6)**	**9112 (4.7)**	**18 625 (5.7)**

## DISCUSSION

Prevalence data are important for the surveillance of overweight and obesity, provided similar cut-off values and references are used consistently. However, there is no consensus on either cut-off values or on the type of reference used to calculate the prevalence of overweight and obesity, which makes it difficult to compare prevalence studies. Until recently, the National Center for Health Statistics/World Health Organization (NCHS/WHO) reference was used,^32^ but one of its most important shortcomings was the lack of a BMI reference for children younger than nine years of age. A revision of the NCHS/WHO reference was conducted by the CDC and recommended as a replacement of the older NCHS/WHO reference.^33^ Another reference was developed based on the analysis of multinational surveys of a large number of children and recommended by the authors for international use.^34^ According to this reference, cut-off points for overweight and obesity in children were obtained by linking the data to the International Obesity Task Force (IOTF) cut-off points for adults (BMI of 25 kg/m^2^ for overweight and 30 kg/m^2^ for obesity). Finally, the WHO, after reviewing data sets from several populations, found that the NCHS data set was the most suitable for a smooth transition with the 2006 WHO child growth standard curves at 5 years with a good alignment with the IOTF cut-off values at 18 years. Accordingly, the WHO reconstructed the 1977 NCHS/WHO reference using state-of-the-art statistical analysis. The result was the development of the 2007 WHO growth reference, which is recommended for international use.^35^

In this analysis, the 2007 WHO reference was chosen for the calculation of prevalence because of the advantages and the potential to be the future international reference for the surveillance of overweight and obesity. In addition, the 2000 CDC reference was chosen to point out the implication of using this reference on prevalence values and to allow for comparison of our results with others that use it as reference, because it is still widely used in many health institutions around the world. Comparison with the other references suggested for international use was not performed in this study^34^ as other reports indicated less sensitivity in the detection of overweight than the 2000 CDC reference.^36^,^37^

Using the 2007 WHO reference, the national prevalence of overweight and obesity in our population was established. Comparison of prevalence data in this report with those from other populations was possible only if similar references and cut-offs were used. To our knowledge, there are no reports on prevalence using the 2007 WHO reference. Therefore, comparisons were made with prevalence rates in reports from other populations using the 2000 CDC reference with cut-off values similar to those used by the WHO. The higher prevalence of overweight and obesity in our sample using the 2007 WHO reference (23.9% and 9.5%, respectively) than that using the 2000 CDC reference (20.4% and 5.7%, respectively) may be explained by the different characterestics of the two references. This suggests that the CDC reference underestimates the prevalence of overweight and obesity and points out the effect of the type of reference used on prevalence data.

In a report from a representative sample of US children collected between 2003 and 2006 using cut-off values similar to those of the WHO, the prevalence of overweight (BMI >85th percentile) and obesity (BMI >97th percentiles) in children 2 to 19 years of age was 31.9% and 11.3%, respectively.^11^ This was much higher than the prevalence rates of 20.4% and 5.7% for overweight and obesity reported in this study. However, the difference is difficult to interpret because of the different age groups between the two studies. In a report from Mexico, in a nationally representative sample of 10- to 17-year-old children collected in the year 2000, there was a higher prevalence of excess weight in girls than in boys (14.3%-19.1% vs 10.8%-16%).^14^ Our findings were consistent with those of this report although our prevalence levels were higher (25.1% vs 24.7%) than those reported from Mexico. This difference may be due to time factors (4-5 years' difference in data collection) or a truly higher prevalence in our populations.^14^ It seems that the prevalence of overweight in our population is intermediate between those of the US and Mexico. The overall pattern of gender variation in the prevalence of overweight and obesity from 5-18 years of age, indicating a higher prevalence of overweight in girls, is consistant with patterns reported from Mexico, UAE, and Saudi Arabia.^14^,^17^,^22^ However, the opposite pattern (higher prevalence in boys) was true for the prevalence of obesity and severe obesity.

In conclusion, this report establishes baseline national prevalence rates of overweight, obesity and extreme obesity in Saudi school-age children and adolescents, indicating intermediate levels between developing and industrialized countries. Preventive measures should be instituted by health authorities to prevent further increases in the prevalence of overweight in school-age children and adolescents and the associated health hazards.
