# errata

**Published:** 2010

El Mouzan MI, Foster PJ, Al Herbish AS, Al Salloum AA, Al Omer AA, Qurachi MM, Kecojevic T. Prevalence of overweight and obesity in Saudi children and adolescents. Ann Saudi Med 2010;30(3):203-208. PMID: 20427936. Some data in some of the tables (and the corresponding data in the text) are corrected as indicated. Changes are highlighted.

**Table 1:** Prevalence of overweight and obesity.

Age (years)	BMI> +1 SDS Number of children (%)			BMI> +2 SDS Number of children (%)			BMI> +3 SDS Number of children (%)		
	Boys	Girls	All	Boys	Girls	All	Boys	Girls	All
5–12	6149 (19.9)	5917 (19.2)	12066 (19.6)	6149 (9)	5917 (6.8)	12 066 (7.9)	6149 (2)	5917 (1)	12 066 (1.5)
13–18	3659 (24.8)	3592 (28.4)	7251 (26.6)	3659 (11.2)	3592 (10.0)	7251 (10.6)	3659 (2.6)	3592 (2.1)	7251 (2.4)
**Overall**	**9808** (21.7)	**9509** (22.7)	**19317** (22.2)	**9808** (9.7)	**9509** (8.0)	**19 317** (8.9)	**9808(2.3)**	**9509 (1.6)**	**19317 (2.0)**

SDS: standard deviation scores

**Table 4:** Comparative prevalence of overweight using the WHO and CDC references.

Age (years)	2007 WHO Number of children (%)			2000 CDC Number of children (%)		
	Boys	Girls	All	Boys	Girls	All
5–12	6149 (19.9)	5917 (19.2)	12066 (19.6)	6260 (15.9)	5985 (15.7)	12245 (15.8)
13–18	3659 (24.8)	3592 (28.4)	7251 (26.6)	3253 (24.7)	3127 (25.1)	6380 (24.9)
**Total**	**9808** (21.7)^a^	**9509** (22.7)^a^	**19317** (22.2)^b^	9513 (18.2)^a^	**9112** (18.9)^a^	18 625 (18.6)^b^

a No statistically significant difference between boys and girls,

b Statistically significant higher prevalence of overweight using the WHO reference (*P*<.0001) (The 2000 CDC reference underestimates the prevalence of overweight).

**Table 5:** Comparative prevalence of obesity using the WHO and CDC references.

Age (years)	2007 WHO Number of children			2000 CDC Number of children (%)		
	Boys	Girls	All	Boys	Girls	All
5–12	6149 (9)	5917 (6.8)	12066 (7.9)	6260 (5)	5985 (3.9)	12245 (4.5)
13–18	3659 (11.2)	3592 (10)	7251 (10.6)	3253 (8.2)	3127 (5.5)	6380 (7)
**Total**	**9808** (9.7)^a^	**9509** (8.0)^a^	**19317** (8.9)^b^	**9513** (6.0)^a^	**9112** (4.7)^a^	**18625** (5.4)^b^

a No statistically significant difference between boys and girls,

b Statistically significant higher prevalence of overweight using the WHO reference (*P*<.0001) (The 2000 CDC reference underestimates the prevalence of obesity).

